# Development of dim-light vision in the nocturnal reef fish family Holocentridae. I: Retinal gene expression

**DOI:** 10.1242/jeb.244513

**Published:** 2022-09-08

**Authors:** Lily G. Fogg, Fabio Cortesi, David Lecchini, Camille Gache, N. Justin Marshall, Fanny de Busserolles

**Affiliations:** 1 Queensland Brain Institute, The University of Queensland, Brisbane, Queensland 4072, Australia; 2 PSL Research University, EPHE-UPVD-CNRS, UAR3278 CRIOBE, 98729 Papetoai, Moorea, French Polynesia; 3 Laboratoire d'Excellence “CORAIL”, Paris 75006, France

**Keywords:** Ontogeny, Multibank retina, Teleost fish, Opsins, Transcriptomics

## Abstract

Developmental changes to the visual systems of animals are often associated with ecological shifts. Reef fishes experience a change in habitat between larval life in the shallow open ocean to juvenile and adult life on the reef. Some species also change their lifestyle over this period and become nocturnal. While these ecological transitions are well documented, little is known about the ontogeny of nocturnal reef fish vision. Here, we used transcriptomics to investigate visual development in 12 representative species from both subfamilies, Holocentrinae (squirrelfishes) and Myripristinae (soldierfishes), in the nocturnal coral reef fish family, Holocentridae. Results revealed that the visual systems of holocentrids are initially well adapted to photopic conditions with pre-settlement larvae having high levels of cone opsin gene expression and a broad cone opsin gene repertoire (8 genes). At reef settlement, holocentrids started to invest more in their scotopic visual system, and compared with adults, showed upregulation of genes involved in cell differentiation/proliferation. By adulthood, holocentrids had well developed scotopic vision with high levels of rod opsin gene expression, reduced cone opsin gene expression and repertoire (1–4 genes) and upregulated phototransduction genes. Finally, although the two subfamilies shared similar ecologies across development, their visual systems diverged after settlement, with Myripristinae investing more in scotopic vision than Holocentrinae. Hence, both ecology and phylogeny are likely to determine the development of the holocentrid visual system.

## Abstract (precis)

**Summary:** Coral reef fishes in the family Holocentridae remodel their retina at the molecular level to adapt to a nocturnal lifestyle during development.

## INTRODUCTION

Vision underlies many behaviours crucial to survival, most notably foraging, mating and predator avoidance (Cronin et al., 2014). As a result of their varied ecologies and the different light environments that they inhabit, marine fishes possess exceptionally diverse visual adaptations. These adaptations are reflected at the molecular level in the class, copy number and level of expression of the genes in their retina (de Busserolles et al., 2020; Cortesi et al., 2020). The retina has two main types of light-sensing cells: rods and cones (Lamb, 2013). Rods usually contain the visual pigment, rhodopsin (RH1, rhodopsin-like middle-wavelength sensitive 1) and mediate scotopic (dim light) vision. Cones mediate photopic (bright light) and colour vision and are divided into single and double cones (i.e. two fused single cones). Single cones usually have SWS1 (short-wavelength sensitive 1) and SWS2 opsins, while double cones usually have RH2 (rhodopsin-like middle-wavelength sensitive 2) and LWS (long-wavelength sensitive) opsins (Bowmaker, 2008; Carleton et al., 2008; Dalton et al., 2017; reviewed in Carleton et al., 2020). Each opsin class has a different range of spectral sensitivities, including UV–violet (SWS1), violet–blue (SWS2), blue–green (RH2 and RH1) and green–red (LWS), that can be tuned to match the environmental light conditions (Lythgoe, 1979; Cronin et al., 2014; Schweikert et al., 2018; reviewed in Carleton et al., 2020; Musilova et al., 2021).

Interspecific differences in many retinal genes, particularly changes in the expression of the opsin genes and their spectral sensitivities, correlate well with ecological demands (Shand, 1997; Stieb et al., 2016; Luehrmann et al., 2020). For instance, fishes living in dim environments (e.g. deep-sea habitat or nocturnal lifestyle) have evolved a shared array of molecular adaptations to enhance the sensitivity of their eyes. These include high levels of *rh1* gene expression and low levels of cone opsin gene expression (Luehrmann et al., 2019; de Busserolles et al., 2021; Lupše et al., 2021), and spectral tuning of their *rh1* gene to wavelengths that predominate in their depth range (Toller, 1996; Douglas et al., 2003). Furthermore, several species show more extreme scotopic adaptations, such as exclusive expression of rod-specific genes (Lupše et al., 2021) and duplication of their *rh1* gene (Musilova et al., 2019). Despite the fact that many of these adaptations are relatively widespread, little is known about their development.

During development, most marine fishes undergo significant shifts in ecology. Larval marine fishes typically inhabit the (zoo)plankton-rich upper layers of the epipelagic ocean (Job and Bellwood, 2000; Helfman et al., 2009), where the available light is bright and broad spectrum, ranging from UV (<400 nm) to red (>600 nm) (Boehlert, 1996). However, as juveniles and adults, different species may transition to very different habitats (pelagic, estuarine, reef, deep-sea) and adopt contrasting diets (planktivory, carnivory, herbivory, corallivory), and diel activity patterns (diurnal, nocturnal, crepuscular) (King and McFarlane, 2003; Helfman et al., 2009). These changes in light environment and ecological demands are thought to be the main drivers of visual system development in marine fishes (Carleton et al., 2020; Musilova et al., 2021). Accordingly, molecular changes in their visual systems have previously been correlated with ontogenetic changes in diet (surgeonfishes: Tettamanti et al., 2019), depth (numerous deep-sea species: Lupše et al., 2021), habitat (clown anemonefish: Roux et al., 2022 preprint) and colouration (dottybacks: Cortesi et al., 2016).

In marine fishes that adopt bright environments, visual development is characterised by typical changes to the retina at the molecular level. In general, the retina is dominated by cone opsin gene expression at early larval stages, while the onset of *rh1* expression is initially delayed but subsequently increases to become the dominantly expressed opsin gene in the post-metamorphic retina (Helvik et al., 2001; Mader and Cameron, 2004; Tettamanti et al., 2019; Wang et al., 2021; Frau et al., 2022). This overall shift is accompanied by more species-specific modifications, such as increased expression of cone opsin genes that are sensitive to ecologically relevant wavelengths (Shand et al., 2008; Cortesi et al., 2016; Savelli et al., 2018; Tettamanti et al., 2019). In contrast, visual development in fishes that adopt dim environments seems to be characterised by a more rapid and extreme version of these changes. For example, some deep-sea fishes express cone opsin genes as larvae but progress to expressing only rod opsin genes in adulthood (Bozzano et al., 2007; de Busserolles et al., 2014; Lupše et al., 2021). However, most of the previous studies on visual development in dim environments focused on deep-sea fishes (Cortesi et al., 2020). Conversely, very little is known about how the visual system develops in nocturnal reef fishes (but see Job and Shand, 2001).

Here, we investigated visual development at the molecular level in the nocturnal reef fish family, Holocentridae. Larvae from both subfamilies, Holocentrinae (squirrelfishes) and Myripristinae (soldierfishes), inhabit the upper pelagic ocean where they feed on zooplankton (Tyler et al., 1993; Sampey et al., 2007). Around metamorphosis, most holocentrids (except for a few species that migrate to deeper waters) migrate to a shallow tropical coral reef habitat (Nelson, 1994) and adopt a nocturnal lifestyle feeding on benthic crustaceans (Holocentrinae) or zooplankton in the water column (Myripristinae) (Gladfelter and Johnson, 1983; Greenfield, 2002*,* 2017). We recently characterised the visual systems of adult holocentrids (de Busserolles et al., 2021) and demonstrated strong adaptation for scotopic vision, with *rh1*-dominated opsin gene expression. Notably, their rod spectral sensitivities have been shown to be tuned to their preferred depth, i.e. blue-shifted when living deeper compared with red-shifted in the shallows (Toller, 1996; Yokoyama and Takenaka, 2004). Finally, adults also have some degree of photopic vision – more so in Holocentrinae than Myripristinae – with the presence of single cones expressing *sws2* and double cones expressing one or two *rh2* genes (de Busserolles et al., 2021).

Although the visual systems of adult holocentrids have been well characterised, little is known about how they develop. Thus, we used high-throughput RNA sequencing to examine opsin gene expression and whole-transcriptome differential gene expression in the retina at key ontogenetic stages (pre-settlement larvae, settlement larvae, settled juveniles and adults). We studied shallow reef-dwelling species from three genera (*Sargocentron*, *Neoniphon* and *Myripristis*) spanning both holocentrid subfamilies, as well as an adult of deeper-dwelling species (*Ostichthys* sp.). Using this approach, we aimed to assess how the holocentrid visual system changes on a molecular level as they shift from a bright to a dim environment.

## MATERIALS AND METHODS

### Animal collection and retinal tissue preservation

Details of all animals used in this study are given in Table S1. Pre-settlement larvae, which are pelagic larvae close to transitioning to the reef, were collected on the Great Barrier Reef around Lizard Island, Australia (14°40′S, 145°27′E) using light traps in February 2020. Settlement larvae, larvae that have just transitioned to the reef, were collected using a crest net positioned on the reef crest of the lagoon at Temae, Moorea, French Polynesia (17°29'S, 149°45'W) in February and March 2019 (Lecchini et al., 2004; Besson et al., 2017). Settled juveniles were larvae caught in light traps on Lizard Island which were allowed to metamorphose and further develop for 2 weeks in outdoor aquaria exposed to natural light in March 2017. Adults were collected with either spearguns or pole and lines on the reefs surrounding Moorea in March 2018 and 2019 or collected with clove oil and hand nets at Lizard Island in February 2020. Some adults were also sourced from a supplier, Cairns Marine (Cairns Marine Pty Ltd, Cairns, Australia; https://www.cairnsmarine.com/), that collects from the northern Great Barrier Reef.

Fish collection and euthanasia followed procedures approved by the University of Queensland Animal Ethics Committee (QBI 304/16). Briefly, fish were first anesthetised by immersion in a solution of 0.2 ml of clove oil per litre of seawater until respiration and all response to light and touch had ceased and were then euthanised by swift decapitation. All collections within Australia were conducted under a Great Barrier Reef Marine Park Permit (G17/38160.1) and Queensland General Fisheries Permit (180731) and all collections in French Polynesia were conducted in accordance with French regulations. Following euthanasia, all individuals were photographed with a ruler and their body size [total length and standard length] and eye diameter subsequently measured from photographs using Fiji v1.53c (National Institutes of Health, USA; Schindelin et al., 2012). Eyes were immediately enucleated, the cornea and lens removed, and the eye cup preserved in RNAlater (Sigma-Aldrich).

### Transcriptome sequencing, quality control and *de novo* assembly

Retinal transcriptomes were sequenced for a total of 22 individuals in Holocentridae: two pre-settlement larvae (*Sargocentron rubrum, n=*2), seven settlement larvae (*Sargocentron punctatissimum*, *n*=4; *Myripristis berndti*, *n*=1; *Myripristis pralinia*, *n*=1; *Myripristis kuntee*, *n*=1), six settled juveniles (*S. rubrum, n*=3; *Sargocentron cornutum*, *n*=1; *Neoniphon sammara*, *n*=2) and seven adults (*S. punctatissimum*, *n*=3; *S. rubrum*, *n*=2; *M. kuntee*, *n*=1; *Ostichthys* sp., *n*=1). The adult dataset was completed with previously published transcriptomic (*S. rubrum*, *n*=1; *Sargocentron spiniferum*, *n*=1; *Sargocentron diadema*, *n*=1; *N. sammara*, *n*=3; *M. berndti*, *n*=4; *Myripristis jacobus*, *n*=2, *Myripristis murdjan*, *n*=1) and genomic (*M. jacobus*, *n*=1) data (Malmstrøm et al., 2017; Musilova et al., 2019; de Busserolles et al., 2021), resulting in a total dataset of 35 retinal transcriptomes and one genome, spanning 12 species.

Briefly, initial retinal tissue digestion was conducted using Proteinase K (20 mg ml^−1^, 15–30 min digest at 55°C; New England Biolabs). Total RNA was extracted and isolated from the retinas using the Monarch Total RNA Miniprep Kit (New England Biolabs) and genomic DNA was removed using RNase-free DNase (Monarch). Quality and yield of isolated RNA was assessed using the Eukaryotic Total RNA 6000 Nano kit (Agilent technologies) and the Queensland Brain Institute's Bioanalyser 2100 (Agilent technologies). RNA extractions were shipped on dry ice and whole-retina transcriptome libraries were prepared from total RNA using the NEBNext Ultra RNA library preparation kit for Illumina (New England Biolabs) at Novogene's sequencing facilities in Beijing, Hong Kong and Singapore. The concentration of each library was checked using a Qubit dsDNA BR Assay kit (Thermo Fisher) prior to barcoding and pooling at equimolar ratios. Libraries were sequenced as 150 bp paired-end reads on a HiSeq 2500 using Illumina's SBS chemistry version 4. Libraries were trimmed and *de novo* assembled as described in de Busserolles et al. (2017). Briefly, read quality was assessed using FastQC (v.0.72), raw reads were trimmed and filtered using Trimmomatic (v.0.36.6) and transcriptomes were *de novo* assembled with Trinity (v.2.8.4) using the genomics virtual laboratory on the Galaxy platform (usegalaxy.org; Afgan et al., 2018).

### Opsin gene mining, phylogenetic reconstruction and expression analyses

All cytochrome c oxidase subunit I (COI) and opsin gene extractions and expression analyses were conducted in Geneious Prime v.2021.1.1 (Biomatters Ltd). Initially, COI genes were extracted from *de novo* assembled transcriptomes for species identification by mapping to species-specific references from GenBank (https://www.ncbi.nlm.nih.gov/genbank/) with medium sensitivity settings. Opsin gene extractions were performed by mapping assembled transcriptomes to published opsin coding sequences (CDS) of the dusky dottyback (*Pseudochromis fuscus)* (Cortesi et al., 2016) with customised sensitivity settings (fine tuning, none; maximum gap per read, 15%; word length, 14; maximum mismatches per read, 40%; maximum gap size, 50 bp; index word length, 12; paired reads must both map). Contigs mapped to COI and opsin references were scored for similarity against publicly available sequences using BLASTn (NCBI, Bethesda, MD; https://blast.ncbi.nlm.nih.gov/Blast.cgi). One of the limitations of *de novo* assembly of highly similar genes (such as opsin gene paralogs) using short-read transcripts is that it can produce erroneous (chimeric) sequences or fail to reconstruct lowly expressed transcripts. Thus, a second approach was also employed using a manual extraction method from back-mapped reads to verify the initially extracted opsin genes, as per de Busserolles et al. (2017).

During manual gene extraction, filtered paired reads were mapped against *P. fuscus* reference opsin CDS (with previously stated customised sensitivity settings) in Geneious Prime. Matching reads were connected by following single nucleotide polymorphisms (SNPs) across genes with continual visual inspection for ambiguity and were extracted as paired mates to mitigate sequence gaps. The consensus of an assembly of these extracted reads was used as the reference for low sensitivity (high accuracy, 100% identity threshold) mapping. Partial CDS extractions were cyclically mapped using the low sensitivity approach to prolong and subsequently remap reads until a complete CDS was obtained.

To confirm the identity of each gene, full coding sequences were preliminarily checked on BLASTn and then used in conjunction with a reference dataset obtained from GenBank (www.ncbi.nlm.nih.gov/genbank/) and Ensembl (www.ensembl.org/) to reconstruct the opsin gene phylogeny (de Busserolles et al., 2017*,* 2021). All opsin gene sequences were aligned using the MUSCLE plugin v.3.8.425 (Edgar, 2004) in Geneious Prime. MrBayes v.3.2.6 (Ronquist et al., 2012) on CIPRES (Miller et al., 2010) was used to reconstruct a phylogenetic tree from the aligned sequences using the following parameters: GTR+I+G model, two independent MCMC searches with four chains each, 10 million generations per run, 1000 generations sample frequency and 25% burn-in. The generated tree was edited in Figtree v.1.4.4 (http://tree.bio.ed.ac.uk/software/figtree/).

For differential expression analyses, opsin gene paralogs were first scored on similarity using pairwise/multiple alignments. The similarity score minus 1 was used as the gene-specific maximum percentage mismatch threshold for mapping (paired) transcripts back onto complete extracted opsin CDS to ensure that reads did not map to multiple paralogs. The proportional expression of rod versus cone opsin genes was calculated as the number of reads mapped to either *rh1* or all cone genes divided by the number of reads mapped to all genes, adjusted to account for differing gene lengths. The proportional expression of single versus double cone opsin genes was calculated as the number of reads mapped to each single (i.e. *sws1* and *sws2*) or double (i.e. *rh2* and *lws*) cone opsin gene copy divided by the number of reads mapped to all single or double cone opsin genes, respectively and adjusted for gene length.

### Whole-transcriptome differential gene expression analyses

Further analyses were conducted to search for differentially expressed genes (DEGs) over development across the whole retinal transcriptome for *S. punctatissimum* (settlement larvae, *n*=3; adults, *n*=3). To control for diel fluctuations in visual gene expression, all individuals were euthanised at 08:30 h (Yourick et al., 2019). All analyses were conducted using the genomics virtual laboratory on the Galaxy platform. Firstly, the quality of the sequencing reads was assessed using FastQC (v.0.72) and raw reads were subsequently trimmed and filtered using Trimmomatic (v.0.36.6), as described previously. Given that a high-quality reference genome was not available for the species sequenced, a reference transcriptome was *de novo* assembled using Trinity (v.2.8.4) from the combined reads of one paired-end library from each life stage (with settings described above). A mapping-based estimation of transcript abundance was then obtained using Salmon quant v.0.14.1.2 (using default settings except specifying paired-end, stranded reads (SF), discarding orphan quasi, validating mappings and mimicking Bowtie) (Patro et al., 2017). Differentially expressed transcripts were identified using DEseq2 v.2.11.40.6 (using default settings with the following changes: setting input data to transcripts per kilobase million (TPM) values generated by Salmon, outputting normalised counts table and no independent filtering) (Love et al., 2014). The DEseq2 result file was filtered (Filter v.1.1.0) on the adjusted *P*-value column (≤0.05) to obtain DEGs.

Separate lists of up- and down-regulated DEGs were obtained by filtering for positive and negative values in the log fold change column of the DEseq2 result file. These lists were re-formatted (using FASTA-to-TABULAR v.1.1.1, Cut v.1.0.2, Sort v.1.1.1, Join v.1.1.2 and TABULAR-to-FASTA v.1.1.1 tools) to generate simple gene ID-to-sequence lists of DEGs in FASTA format. The top BLAST hit from the UniProtKB/Swiss-Prot database (The UniProt Consortium, 2018) was obtained using NCBI BLAST+ blastx v.0.3.3 (using default settings but selecting blastx analysis) and Blast top hit descriptions v.0.0.9, and was used to annotate DEG sequences (Altschul et al., 1997; Camacho et al., 2009; Cock et al., 2013*,* 2015). For the top 15 DEGs, QuickGO (Binns et al., 2009) and UniProtKB were used to manually search for gene ontology (GO) and function. For lists of DEGs upregulated at each life stage, PANTHER 17.0 (http://www.pantherdb.org/; Mi et al., 2021) was used via The Gene Ontology Resource website to perform a GO statistical overrepresentation analysis for biological processes (Ashburner et al., 2000; Gene Ontology Consortium, 2021). PANTHER analyses used *Oryzias latipes* as the reference, used Fisher's exact test, calculated false discovery rate (FDR), used an FDR-adjusted *P*-value of <0.05 and filtered results for GO terms with fold enrichment ≥6 (i.e. highly overrepresented).

## RESULTS

### Opsin gene expression

**Fig. 1.**
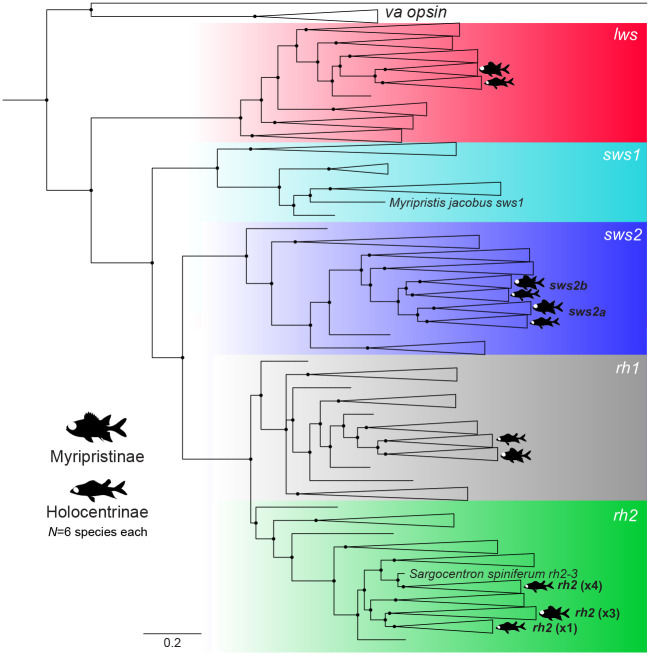
**Vertebrate opsin gene phylogeny.** Phylogeny including opsin genes from 6 species per subfamily in Holocentridae denoted as per legend. All expressed genes fell into four out of the five main classes (Bowmaker, 2008), while the only *sws1* sequence was derived from a genome (Musilova et al., 2019). Numbers in brackets are number of *rh2* paralogs. Black circles denote Bayesian posterior probabilities >0.8. The scale bar denotes substitutions per site. *rh1*, rhodopsin-like middle-wavelength sensitive 1 (rod opsin); *rh2*, rhodopsin-like middle-wavelength sensitive 2; *sws1*, short-wavelength-sensitive 1; *sws2*, short-wavelength-sensitive 2; *lws*, long-wavelength-sensitive; *va opsin*, vertebrate ancient opsin (outgroup). GenBank accession numbers are provided in Table S2.

**Fig. 2.**
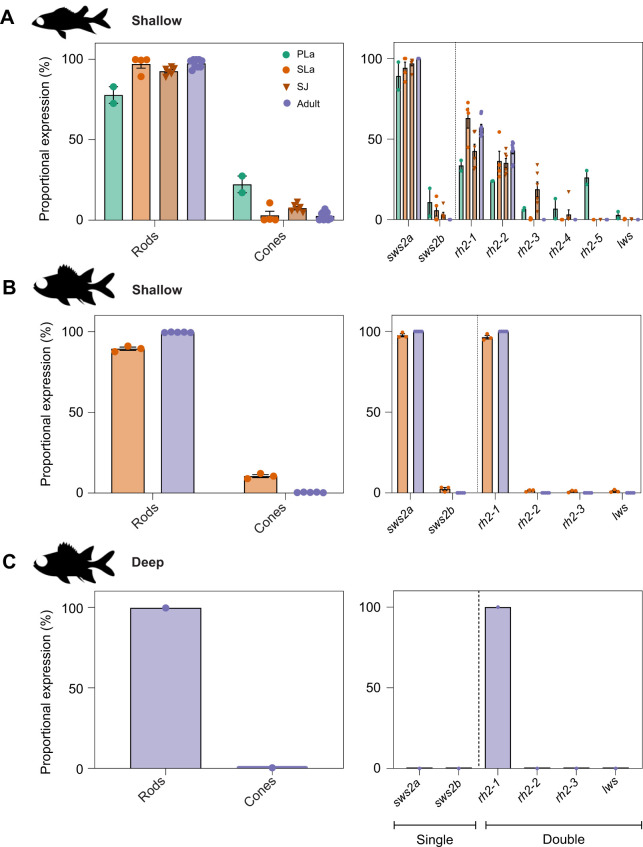
**Opsin gene expression in holocentrids throughout ontogeny.** Proportional rod opsin gene expression (given as % of total opsin gene expression) and proportional cone opsin gene expression [as % of single (*sws* genes) or double (*rh2* genes and *lws*) cone opsin gene expression] in (A) Shallow-dwelling Holocentrinae [pre-settlement larvae (*n*=2), settlement larvae (*n*=4), settled juveniles (*n*=6) and adults (*n*=9) from *Sargocentron rubrum, S. punctatissimum*, *S. cornutum*, *Neoniphon sammara*] (note that all species in the subfamily Holocentrinae are shallow dwelling), (B) shallow-dwelling Myripristinae [settlement larvae (*n*=3) and adults (*n*=5) from *Myripristis berndti, M. kuntee,* and *M. pralinia*] and (C) deep-dwelling Myripristinae [adults (*n*=1) from *Ostichthys* sp.]. Data are means±s.e.m. Green, pre-settlement larvae (PLa); light orange, settlement-stage larvae (SLa); dark orange, settled juveniles (SJ); purple, adults. *rh1*, rhodopsin-like middle-wavelength sensitive 1 (rod opsin); *rh2*, rhodopsin-like middle-wavelength sensitive 2; *sws2*, short-wavelength-sensitive 2; *lws*, long-wavelength-sensitive.

Opsin gene expression was analysed across ontogeny in 8 species in Holocentridae (4 species per subfamily). Phylogenetic reconstruction resolved the class of each opsin gene (Fig. 1) and quantitative opsin gene expression analyses revealed stage-specific expression patterns (Fig. 2, Table 1). At pre-settlement, *S. rubrum* (the only species obtained at this stage) expressed one rod opsin and 8 cone opsins: the rod-specific *rh1* (mean±s.e.m., 78±5% of total opsin gene expression)*,* violet-blue-sensitive *sws2a* (89±9% of single cone opsin gene expression) and *sws2b* (11±9% of single cone opsin gene expression), 5 green-sensitive *rh2s* (*rh2-1*–*rh2-5*: 7±1 to 34±3% of double cone opsin gene expression) and red-sensitive *lws* (3±2% of double cone opsin gene expression).

**Table 1.**
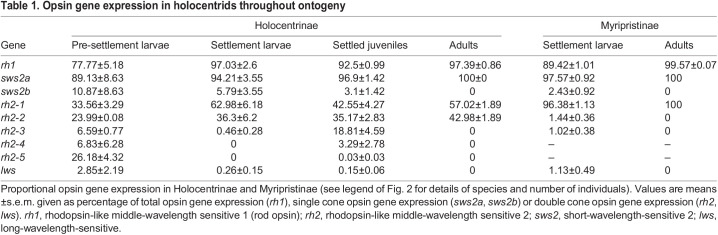
Opsin gene expression in holocentrids throughout ontogeny

At settlement, all species in Holocentridae expressed one rod opsin and 6–8 cone opsins: an *rh1* (Holocentrinae: 97±3%, Myripristinae: 89±1%), *sws2a* (Holocentrinae: 94.2±3.6%; Myripristinae: 97.6±1%), *sws2b* (Holocentrinae: 5.8±3.6%; Myripristinae: 2.4±1%), *lws* (Holocentrinae: 0.3±0.16%; Myripristinae: 1.1±0.5%) and several *rh2* paralogs (Holocentrinae: 63±6%; Myripristinae: 96.4±1%; % for the most highly expressed paralog, *rh2-1*) (Fig. 2, Table 1). Most Holocentrinae species expressed 4 or 5 *rh2* paralogs at settlement, and one of these was expressed at low levels (≤0.5%) in every species. All Myripristinae species expressed 3 *rh2* paralogs at settlement, with 2 of these expressed at low levels (≤1.5% per paralog). Between settlement and adulthood, all holocentrids increased rod opsin gene expression, decreased cone opsin gene expression, and stopped expressing 3–4 cone opsin genes (*sws2b*, *lws* and 1–2 *rh2* genes).

As adults, all shallow-water holocentrids retained some similarities in their opsin gene repertoires, expressing one rod opsin and two to three cone opsins. All species expressed an *rh1* (Holocentrinae: 97.4±0.9%; Myripristinae: 99.6±0.1%) and *sws2a* (100% in both subfamilies) opsin gene, while the variation in *rh2* paralogs remained, with two *rh2* paralogs expressed in Holocentrinae (*rh2-1*: 57±2%, *rh2-2*: 43±2%) and only one in Myripristinae (100%) (Fig. 2, Table 1). Finally, an adult of the deeper-dwelling species from Myripristinae (*Ostichthys* sp.) expressed a simpler opsin gene repertoire than the shallow-water species, with one *rh1* gene (99.6%) and one *rh2* paralog (100%). Overall, opsin gene expression differed between the subfamilies across development, with differences in per-gene expression levels and the number of opsin classes and *rh2* paralogs expressed. Most notably, Myripristinae showed a greater increase in rod opsin gene expression post-settlement than Holocentrinae and expressed one less *rh2* paralog upon maturity.

### Whole-retina differential gene expression

**Fig. 3.**
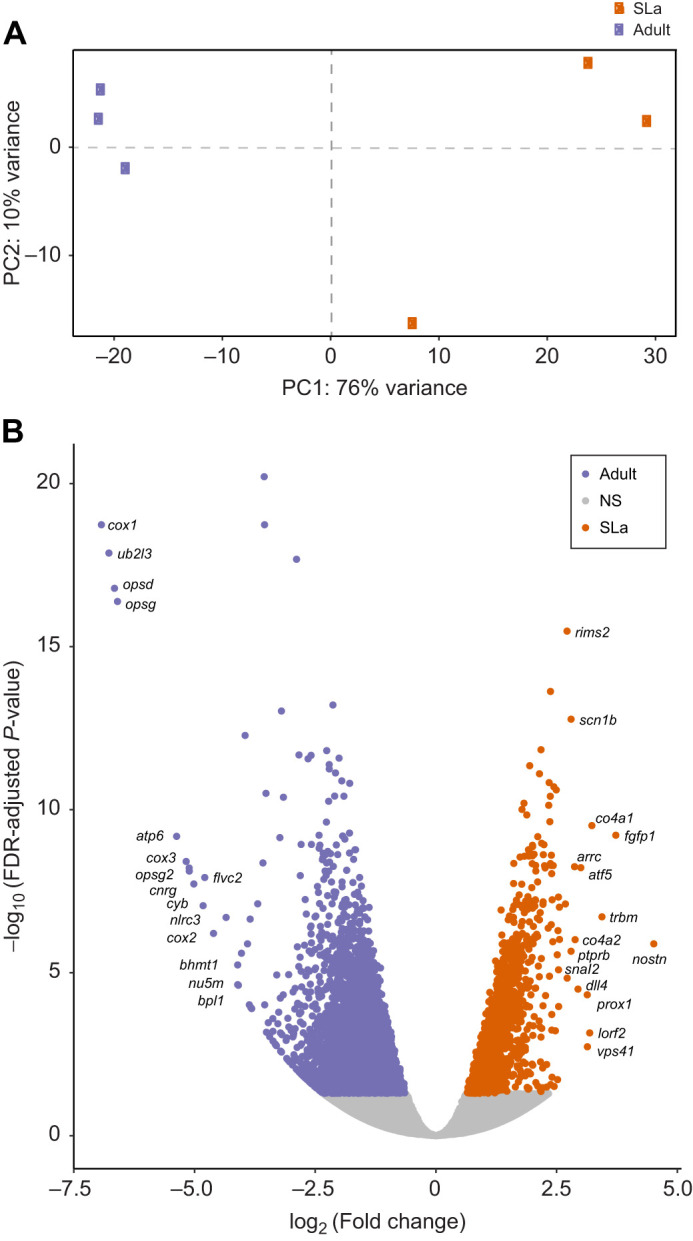
**Differential gene expression in *Sargocentron punctatissimum* throughout ontogeny.** (A,B) Differential retinal gene expression between settlement larvae (SLa; *n*=3) and adult (*n*=3) *S. punctatissimum* (Holocentrinae)*.* (A) PCA plot of rlog-transformed gene counts showing the variance between individual transcriptomes used for differential gene expression analyses. (B) Volcano plot depicting (log_2_) fold changes in expression of all retinal genes against the (−log10) adjusted *P*-value. The top 15 upregulated genes (i.e. greatest fold change; see also Table 2) at each life stage are labelled. Orange dots with a log fold change >0 represent transcripts that were expressed at significantly higher levels in settlement larvae, and purple dots with a log fold change <0 were expressed at significantly higher levels in adults. Grey dots (NS, not significant) represent transcripts that were not differentially expressed.

Differential gene expression across the entire retinal transcriptome was examined during development for *S. punctatissimum*. Transcriptomes separated distinctly by life stage in a PCA (Fig. 3A). Whole-transcriptome analyses revealed that a total of 8395 out of 54,094 transcripts were differentially expressed over ontogeny (adjusted *P*-value<0.05). Upon annotation, this total was refined to 4637 differentially expressed genes (DEGs) (i.e. 8.6% of transcripts). Of the DEGs, 1394 were upregulated in settlement larvae (30.1%) and 3243 were upregulated in adults (69.9%) (Fig. 3B). The top 15 DEGs upregulated in larval retinas were largely involved in cell differentiation/proliferation and cellular structure. Conversely, the top 15 DEGs upregulated in adults were primarily involved in visual perception and aerobic respiration (Table 2). Notably, the DEGs also included several developmental transcription factors (TFs) (Fogg and de Busserolles, 2022). In the larvae, this included upregulation of *otx2* and *otx5*, while in adults, the TFs, *nr2e3* and *rorb* were upregulated.

**Table 2.**
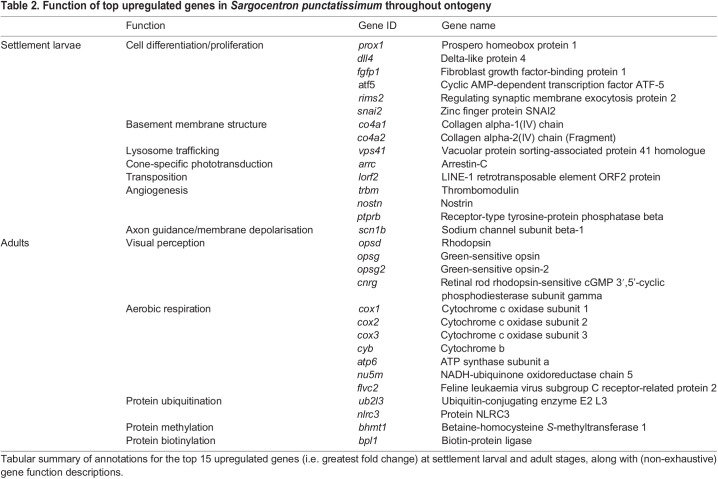
Function of top upregulated genes in *Sargocentron punctatissimum* throughout ontogeny

During development, 5 GO terms were found to be highly enriched in settlement larvae (Table S3) while 22 were highly enriched in adults (Table S4) (FDR-adjusted *P*-value<0.05 and fold enrichment≥6). All GO terms enriched in larvae were related to development, e.g. cell morphogenesis involved in neuron differentiation (GO:0048667) and neurogenesis (GO:0022008). In contrast, most of the GO terms in adults were related to metabolism, e.g. ATP metabolic process (GO:0046034) and aerobic respiration (GO:0009060). Notably, no terms relating to visual perception were among the most highly enriched GO terms at either stage.

## DISCUSSION

### Opsin gene expression during development

Teleosts are known to tune their spectral sensitivities to the light environment during development by changing their relative opsin gene expression levels and/or by switching between gene classes or copies (Shand et al., 2002; Cheng and Flamarique, 2007; Carleton et al., 2008; Savelli et al., 2018; Musilova et al., 2019). Holocentrids are no exception. Developmental changes in holocentrid opsin gene expression correlated well with their switch to a nocturnal, reef-dwelling lifestyle. For example, holocentrids increased their relative *rh1* expression by nearly 20% during development (Fig. 2, Table 1). Furthermore, they stopped expressing most of their cone opsin genes, only retaining cone opsins sensitive to the mid-range (blue–green; *sws2a* and *rh2*) wavelengths that dominate the reef at night. This is in contrast with changes in most diurnal fishes, which do not reduce the number of cone opsins expressed over ontogeny (Takechi and Kawamura, 2005; Cheng and Flamarique, 2007; Shand et al., 2008; Cortesi et al., 2016; Tettamanti et al., 2019; Chang et al., 2020).

**Fig. 4.**
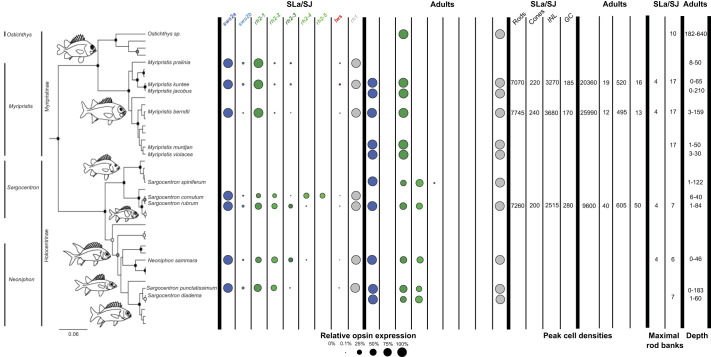
**Summary of visual adaptations in holocentrids during development.** Opsin gene expression, peak retinal cell densities and maximal rod banking in settlement larvae (SLa) or settled juveniles (SJ) and adults (A) alongside depth at maturity (in metres) are overlaid onto the holocentrid phylogeny. Dots represent the expression of opsin genes in the transcriptome with the size of the dot illustrating relative opsin gene expression as a percentage of total (*rh1*), single cone (*sws2a* and *sws2b*) or double cone (*rh2* and *lws*) opsin gene expression. Peak cell densities (given as cells per 0.01 mm^2^ of retina) and maximal rod banking are the highest densities of each respective cell type and the highest number of banks, respectively, found at the given stage after examining the dorsal, ventral, central, nasal and temporal retina. Phylogeny adapted from Dornburg et al. (2012). Note that the *Ostichthys* specimen could not be identified to a species level and so the depth for *Ostichthys kaianus* is shown. The maximal rod banking given for the *Ostichthys* species is an estimation since only ventral sections were available. References for depth: *O. kaianus* (Greenfield et al., 2017), *M. pralinia* (Allen and Steene, 1988), *M. jacobus* (Moore et al., 2015), *M. murdjan* (Lieske and Myers, 1994), *M. violacea* (Allen and Erdmann, 2012), *M. kuntee* (Bacchet et al., 2016), *M. berndti* (Allen and Erdmann, 2012), *S. spiniferum* (Lieske and Myers, 1994), *S. cornutum* (Allen and Erdmann, 2012), *S. punctatissimum* (Lieske and Myers, 1994), *S. diadema* (Randall, 1998), *S. rubrum* (Randall, 1998) and *N. sammara* (Lieske and Myers, 1994). *rh1*, rhodopsin-like middle-wavelength sensitive 1 (rod opsin); *rh2*, rhodopsin-like middle-wavelength sensitive 2; *sws2*, short-wavelength-sensitive 2; *lws*, long-wavelength-sensitive. Morphological data from Fogg et al. (2022).

Among the cone opsin genes, ontogenetic changes in *rh2* expression are particularly interesting in the holocentrids. With 8 copies, holocentrids have the highest number of genomic *rh2* paralogs of any teleost species (Musilova et al., 2019). It should be noted that our phylogeny was insufficiently weighted to resolve some of the *rh2* gene clades (Musilova and Cortesi, 2021 preprint). Nevertheless, our results show that most of these *rh2* paralogs, along with several other cone opsin genes, were only expressed at early life stages (Fig. 2, Table 1). Moreover, some of these genes (e.g. *lws*) were expressed at low levels that may not be functionally relevant to vision. Instead, these paralogs expressed at low levels may have an exclusively developmental function, similar to the sequentially expressed opsins that mediate photoreceptor development in salmonoid and cyprinid fishes (Raymond et al., 1995; Stenkamp et al., 1996; Takechi and Kawamura, 2005; Cheng et al., 2007). However, *rh2* was the only cone opsin gene subclass expressed in all stages/species in Holocentridae (Fig. 4) and this subclass is sensitive to wavelengths present in the light environment of these fishes throughout life. Thus, it is possible that the different *rh2* paralogs serve a visual purpose, allowing the fish to switch between opsin gene palettes during ontogeny for more precise control over spectral tuning (known as subfunctionalisation), as reported for cichlids (Spady et al., 2006).

Although differences in opsin gene expression in teleosts are often explained by the light environment and species-specific ecologies, phylogenetic forces also exert control (Tettamanti et al., 2019; Carleton et al., 2008; Stieb et al., 2016). This may also be the case in holocentrids. Although shallow-dwelling species share a similar light environment at every life stage, the two subfamilies only showed similar opsin gene expression early in life. As adults, shallow-dwelling Myripristinae expressed fewer cone opsin genes and higher *rh1* levels than Holocentrinae. This more extreme adaptation for dim-light vision in shallow-dwelling *Myripristis* spp. may be because they are more closely related to deep-dwelling *Ostichthys* spp., resulting in greater similarity to their deep-water relatives. Notably, this potential phylogenetic inertia did not seem to completely negate the influence of ecological drivers. As such, fewer cone opsins were expressed in an adult from the deeper-dwelling genus *Ostichthys* (Myripristinae) compared with shallow-dwelling Myripristinae representatives, aligning well with the depth-related narrowing of spectral sensitivities observed in other teleosts (Schweikert et al., 2018).

### Transcription factor expression during development

The developmental changes in rod and cone opsin gene expression in the holocentrids were accompanied by stage-specific upregulation of TFs linked to photoreceptor development (Fogg and de Busserolles, 2022). Specifically, the larvae showed upregulation of *otx2* and *otx5*, both of which are linked to the differentiation of photoreceptors (Nishida et al., 2003; Sauka-Spengler et al., 2001; Plouhinec et al., 2005). These findings further validate the ongoing developmental changes occurring in the holocentrid retina at settlement. Conversely, adult holocentrids showed upregulation of *nr2e3* and *rorb*, both of which are involved in the development of rod photoreceptors (Chen et al., 2005; Jia et al., 2009; Xie et al., 2019). The upregulation of TFs involved in rod formation in adults correlates well with the higher rod densities (Fogg et al., 2022) and rod opsin gene expression at this stage. Additionally, *nr2e3* is also known to suppress the expression of numerous cone-specific genes (Chen et al., 2005), and thus, may also play a role in the developmental decrease in cone opsin gene expression in the holocentrids.

It should be noted that several TFs linked to photoreceptor development in other vertebrates (i.e. *nrl*, *thrb*, *six7* and *foxq2*) were not differentially expressed between settlement and adulthood in the holocentrids. The lack of differential expression of *nrl*, a TF necessary for rod specification in mammals and zebrafish (Oel et al., 2020), implies that holocentrids utilise an alternative *nrl*-independent pathway to specify rod fate during ontogeny, similarly to Atlantic cod (Valen et al., 2016). Similarly, the TFs, *thrb*, *six7* and *foxq2*, were not differentially expressed between settlement and adulthood. These TFs have been linked to the development of red (*lws*) cones (Volkov et al., 2020), green (*rh2*) cones (Ogawa et al., 2015) and blue (*sws2*) cones (Ogawa et al., 2021), respectively. Thus, the lack of differential expression of these TFs is not so surprising given that *lws*, *rh2* and *sws2* did not show increased expression between settlement and adulthood in the holocentrids. Although these TFs may still play a role in the development of the different cone subtypes prior to settlement.

### Retinal gene expression during development

Around 8.6% of annotated retinal transcripts were differentially expressed during development in holocentrids (Fig. 3). Although whole-retina changes in gene expression have not been studied in teleosts that undergo major ecological shifts, some insights can be gained from other taxa. For example, butterflies show developmental changes in visual gene expression alongside changes in eye structure and therefore, some of the stage-specific expression differences were attributed to cellular composition (Ernst and Westerman, 2021). Similarly, since the holocentrid retina undergoes cellular remodelling during development (Fogg et al., 2022), it is likely that some of the expression changes simply facilitate these structural changes. Indeed, numerous processes relating to cellular development (e.g. cell morphogenesis and generation of neurons) and several genes involved in eye formation and patterning (e.g. *rx3* and *six3*) (Carl et al., 2002; Loosli et al., 2003; Ogawa et al., 2015) were upregulated in settlement larvae (Table S3; Fogg and de Busserolles, 2022). Nevertheless, similarly to findings in frogs (Valero et al., 2017; Schott et al., 2022), several of the differentially expressed genes were correlated with ecological changes during development. Most notably, genes involved in cone- or rod-based photoreception were upregulated in larval or adult holocentrids, respectively (Fogg and de Busserolles, 2022), aligning with their switch from bright to dim environments.

Finally, the most highly upregulated genes in adults were mainly involved in photoreception and aerobic respiration (Table 2), while most of the GO terms enriched in adults were related to aerobic respiration and metabolism (Table S4). The upregulation of photoreception genes in adults is congruent with their higher photoreceptor densities (Fogg et al., 2022) and implies that adults have a higher investment in vision than larvae. In contrast, the enrichment of genes and processes linked to metabolism may be related to general physiological differences between settlement larvae and adults (e.g. potentially increased vascularisation and therefore, oxygen transport in adults), as previously suggested for frogs (Schott et al., 2022). Indeed, since the retina is one of the most energy-consuming organs in the body (Joyal et al., 2018), the significant changes to its composition that occur during development would likely result in substantial changes to its metabolic demands. Overall, in holocentrids, developmental changes to retinal gene expression align well with the retinal structure and ecological demands of each life stage.

### Conclusion

The holocentrid visual system adapts to life in dim light over ontogeny. At the molecular level, they increase rod opsin gene expression, narrow the cone opsin gene expression repertoire from 8 to 1–4 cone opsins, and shift from enrichment of neurogenesis and cell differentiation/proliferation to phototransduction and metabolism. Together, this suggests that ecology drives visual development in Holocentridae. However, subfamily-specific differences in the degree of scotopic specialisation emerged during development (i.e. higher rod opsin gene expression in Myripristinae) and these were correlated with phylogenetic relatedness to deep-water representatives rather than ecology. This suggests that the development of the holocentrid retina may also be somewhat driven by phylogeny. Future studies on visual development in other nocturnal reef fishes as well as other marine fish families with both shallow- and deep-water forms, such as Anomalopidae (flashlight fishes) and Engraulidae (anchovies), may provide further insights into the ecological and phylogenetic drivers of the development of dim-light vision.

## Supplementary Material

Supplementary information
