# Supplementary material for: Development of dim-light vision in the nocturnal reef fish family Holocentridae. I: Retinal gene expression
Source: J Exp Biol. 2022 Sep 8;225(17):jeb244513. doi: 10.1242/jeb.244513 (PMC9482368; doi:10.1242/jeb.244513)
Supplement: Supplementary information [file jexbio-225-244513-s1.pdf]

Table S1. Details of animals used in study. This study used a total of 35 retinal transcriptomes, each from an individual animal, 22 of which were collected in the current study, 13 of which were collected by Musilova *et al.* (2019) or de Busserolles *et al.* (2021). This study also used one genome from Malmstrøm *et al.* (2017). Locations: LI, Lizard Island; MI, Moorea Island; CM, Cairns Marine; CV, Cape Verde. Analyses: RNA-seq, retinal transcriptome sequenced and opsin gene expression evaluated; DGE, differential gene expression analyses encompassing entire retinal transcriptome; Genome, whole genome sequenced and opsin gene sequences extracted. If standard length or eye used was not recorded for an individual, this is marked as n.a. Sequence read archive (SRA) accession numbers are given for individual transcriptomes.

Species	Life stage	Standard length (cm)	Location	Eye used	Analyses performed	SRA Accession number	Reference
Sargocentron rubrum	Pre-settlement larva	3.1	LI	L	RNA-seq	SRR19793295	This study
	Pre-settlement larva	2.8	LI	L	RNA-seq	SRR19793294	This study
	Settled juvenile	3.0	LI	R+L	RNA-seq	SRR19793283	This study
	Settled juvenile	3.1	LI	R+L	RNA-seq	SRR19793280	This study
	Settled juvenile	3.2	LI	R+L	RNA-seq	SRR19793279	This study
	Adult	14.7	CM	L	RNA-seq	SRR19793278	This study
	Adult	13.4	CM	R	RNA-seq	SRR19793277	This study
	Adult	14.0	LI	L	RNA-seq	SRX9440505	(de Busserolles *et al.* 2021)
Neoniphon sammara	Settled juvenile	n.a.	LI	R+L	RNA-seq	SRR19793276	This study
	Settled juvenile	3.8	LI	R+L	RNA-seq	SRR19793275	This study
	Adult	11.9	LI	R	RNA-seq	SRX5060694	(Musilova *et al.* 2019; de Busserolles *et al.* 2021)
	Adult	11.8	LI	R	RNA-seq	SRX5060695	(Musilova *et al.* 2019; de Busserolles *et al.* 2021)
	Adult	9.2	LI	R	RNA-seq	SRX5060692	(Musilova *et al.* 2019; de Busserolles *et al.* 2021)
Myripristis kuntee	Settlement larva	5.7	MI	L	RNA-seq	SRR19793274	This study
	Adult	13	MI	R	RNA-seq	SRR19793293	This study
Myripristis berndti	Settlement larva	4.9	MI	R	RNA-seq	SRR19793292	This study
	Adult	17.7	LI	R	RNA-seq	SRX5060705	(Musilova *et al.* 2019; de Busserolles *et al.* 2021)
	Adult	20.0	LI	L	RNA-seq	SRX5060696	(Musilova *et al.* 2019; de Busserolles *et al.* 2021)

Journal of Experimental Biology • Supplementary information

	Adult	15.3	LI	L	RNA-seq	SRX5060 738	(Musilova *et al.* 2019; de Busserolles *et al.* 2021)
	Adult	18.2	LI	L	RNA-seq	SRX5060 727	(Musilova *et al.* 2019; de Busserolles *et al.* 2021)
Ostichthys sp.	Adult	20.5	MI	R	RNA-seq	SRR1979 3291	This study
Sargocentron punctatissimum	Settlement larva	5.2	MI	R	RNA-seq, DGE	SRR1979 3290	This study
	Settlement larva	6.2	MI	R	RNA-seq, DGE	SRR1979 3289	This study
	Settlement larva	5.5	MI	L	RNA-seq, DGE	SRR1979 3288	This study
	Settlement larva	5.4	MI	R	RNA-seq, DGE	SRR1979 3287	This study
	Adult	n.a.	MI	n.a.	RNA-seq, DGE	SRR1979 3286	This study
	Adult	n.a.	MI	n.a.	RNA-seq, DGE	SRR1979 3285	This study
	Adult	n.a.	MI	n.a.	RNA-seq, DGE	SRR1979 3284	This study
Sargocentron cornutum	Settled juvenile	2.5	LI	R+L	RNA-seq	SRR1979 3282	This study
Myripristis pralinia	Settlement larva	4.9	MI	R	RNA-seq	SRR1979 3281	This study
Sargocentron diadema	Adult	10.8	LI	R	RNA-seq	SRX9440 506	(de Busserolles *et al.* 2021)
Sargocentron spiniferum	Adult	20.4	LI	R	RNA-seq	SRX9440 504	(de Busserolles *et al.* 2021)
Myripristis murdjan	Adult	14.8	LI	L	RNA-seq	SRX9440 507	(de Busserolles *et al.* 2021)
Myripristis jacobus	Adult	n.a.	CV	n.a.	RNA-seq	SRS4076 665	(Musilova *et al.* 2019; de Busserolles *et al.* 2021)
	Adult	n.a.	CV	n.a.	RNA-seq	SRS4076 643	(Musilova *et al.* 2019; de Busserolles *et al.* 2021)
	Adult	n.a.	n.a.	n.a.	WGS	ERX1545 041	(Malmstrøm *et al.* 2017; Musilova *et al.* 2019)

Journal of Experimental Biology • Supplementary information

Table S2. Genbank accession numbers. Accession numbers for opsin gene coding sequences extracted in this study. All extracted opsin gene sequences were full coding sequences (cds), except those labelled as partial cds.

Species	Opsin	Accession number
Myripristis berndti	RH2-2 (partial cds)	ON817105
	RH2-3 (partial cds)	ON817106
Myripristis kuntee	RH2-1	ON817107
	RH2-2 (partial cds)	ON817108
	RH2-3	ON817109
	RH1	ON817127
	SWS2A	ON817132
Myripristis pralinia	RH2-1	ON817110
	RH2-2	ON817111
	RH2-3	ON817112
	RH1	ON817128
	SWS2A	ON817133
	SWS2B	ON817141
	LWS	ON817136
Neoniphon sammara	RH2-3	ON817113
	RH2-4	ON817114
	SWS2B	ON817142
	LWS (partial cds)	ON817137
Ostichthys sp.	RH2B	ON817115
	RH1	ON817129
Sargocentron cornutum	RH2-1	ON817116
	RH2-2	ON817117
	RH2-3	ON817118
	RH2-4	ON817119
	RH2-5	ON817120
	RH1	ON817130
	SWS2A	ON817134
	SWS2B	ON817143
	LWS	ON817138
Sargocentron rubrum	RH2-3	ON817121
	RH2-4	ON817122
	RH2-5	ON817123
	SWS2B	ON817144
	LWS	ON817139
Sargocentron punctatissimum	RH2-1	ON817124
	RH2-2	ON817125
	RH2-3	ON817126
	RH1	ON817131
	SWS2A	ON817135
	LWS (partial cds)	ON817140
	SWS2B (partial cds)	ON817145

Journal of Experimental Biology • Supplementary information

Table S3. Gene ontology terms overrepresented in the holocentrid retina at settlement. Tabular summary of significantly overrepresented gene ontology terms related to biological processes generated by PANTHER that matched to genes that are differentially expressed in the retina over development in *Sargocentron punctatissimum*. GO terms are given in descending order by fold enrichment. Data derived using *Oryzias latipes* as reference and filtered for terms with FDR-adjusted p-value <0.05 and fold enrichment ≥6. FDR, false discovery rate.

	***O. latipes* - Reference**	***S. punctatissimum***					
**Overrepresented GO biological process (GO ID)**	**Frequency**	**Frequency**	**Expected p-value**	**+/-**	**Fold enrichment**	**Raw p-value**	**FDR-adjusted p-value**
cell morphogenesis involved in differentiation (GO:0000904)	387	7	0.61	+	11.54	2.26E-06	1.94E-02
cell morphogenesis involved in neuron differentiation (GO:0048667)	338	6	0.53	+	11.33	1.44E-05	4.12E-02
cell morphogenesis (GO:0000902)	462	7	0.72	+	9.67	7.09E-06	3.04E-02
generation of neurons (GO:0048699)	731	8	1.15	+	6.98	1.51E-05	3.24E-02
neurogenesis (GO:0022008)	794	8	1.24	+	6.43	2.72E-05	3.89E-02

Journal of Experimental Biology • Supplementary information

Table S4. Gene ontology terms overrepresented in the holocentrid retina in adults. Tabular summary of significantly overrepresented gene ontology terms related to biological processes generated by PANTHER that matched to genes that are differentially expressed in the retina over development in *Sargocentron punctatissimum*. GO terms are given in descending order by fold enrichment. Data derived using *Oryzias latipes* as reference and filtered for terms with FDR-adjusted p-value <0.05 and fold enrichment ≥6. FDR, false discovery rate.

	***O. latipes* - Reference**	***S. punctatissimum***					
**Overrepresented GO biological process (GO ID)**	**Frequency**	**Frequency**	**Expected p-value**	**+/-**	**Fold enrichment**	**Raw p-value**	**FDR-adjusted p-value**
positive regulation of double-strand break repair via homologous recombination (GO:1905168)	1	2	0.01	+	> 100	3.57E-04	3.90E-02
positive regulation of double-strand break repair (GO:2000781)	3	3	0.03	+	90.13	2.55E-05	5.49E-03
electron transport coupled proton transport (GO:0015990)	4	3	0.04	+	67.6	4.43E-05	7.78E-03
energy coupled proton transmembrane transport, against electrochemical gradient (GO:0015988)	4	3	0.04	+	67.6	4.43E-05	7.63E-03
negative regulation of intrinsic apoptotic signaling pathway by p53 class mediator (GO:1902254)	5	3	0.06	+	54.08	7.02E-05	1.16E-02
regulation of intrinsic apoptotic signaling pathway by p53 class mediator (GO:1902253)	6	3	0.07	+	45.07	1.04E-04	1.55E-02
negative regulation of signal transduction by p53 class mediator (GO:1901797)	8	3	0.09	+	33.8	2.02E-04	2.68E-02
mitochondrial electron transport, NADH to ubiquinone (GO:0006120)	17	5	0.19	+	26.51	3.46E-06	1.03E-03
mitochondrial respiratory chain	19	4	0.21	+	18.98	1.06E-04	1.55E-02

Journal of Experimental Biology • Supplementary information

complex I assembly (GO:0032981)							
NADH dehydrogenase complex assembly (GO:0010257)	19	4	0.21	+	18.98	1.06E-04	1.53E-02
ATP synthesis coupled electron transport (GO:0042773)	58	9	0.64	+	13.99	4.93E-08	3.27E-05
aerobic electron transport chain (GO:0019646)	52	8	0.58	+	13.87	2.95E-07	1.16E-04
mitochondrial ATP synthesis coupled electron transport (GO:0042775)	53	8	0.59	+	13.61	3.37E-07	1.26E-04
enteric nervous system development (GO:0048484)	28	4	0.31	+	12.88	4.00E-04	4.20E-02
oxidative phosphorylation (GO:0006119)	64	9	0.71	+	12.68	1.06E-07	5.36E-05
respiratory electron transport chain (GO:0022904)	71	9	0.79	+	11.43	2.36E-07	9.70E-05
aerobic respiration (GO:0009060)	95	11	1.05	+	10.44	2.50E-08	2.15E-05
nerve development (GO:0021675)	46	5	0.51	+	9.8	2.38E-04	3.06E-02
electron transport chain (GO:0022900)	88	9	0.98	+	9.22	1.24E-06	4.27E-04
cellular respiration (GO:0045333)	110	11	1.22	+	9.01	9.91E-08	5.34E-05
ATP metabolic process (GO:0046034)	130	12	1.44	+	8.32	5.83E-08	3.59E-05
energy derivation by oxidation of organic compounds (GO:0015980)	140	12	1.55	+	7.73	1.24E-07	5.92E-05

Journal of Experimental Biology • Supplementary information

References

de Busserolles, F., Cortesi, F., Fogg, L., Stieb, S. M., Luehrmann, M., and Marshall, N. J. 2021. 'The visual ecology of Holocentridae, a nocturnal coral reef fish family with a deep-sea-like multibank retina', *J Exp Biol*, **224**, jeb233098.

Malmstrøm, M., Matschiner, M., Tørresen, O. K., Jakobsen, K. S., and Jentoft, S. 2017. 'Whole genome sequencing data and de novo draft assemblies for 66 teleost species', *Scientific Data*, **4**, 160132.

Musilova, Z., Cortesi, F., Matschiner, M., Davies, W. I. L., Patel, J. S., Stieb, S. M., de Busserolles, F., Malmstrom, M., Torresen, O. K., Brown, C. J., Mountford, J. K., Hanel, R., Stenkamp, D. L., Jakobsen, K. S., Carleton, K. L., Jentoft, S., Marshall, J., and Salzburger, W. 2019. 'Vision using multiple distinct rod opsins in deep-sea fishes', *Science*, **364**, 588-92.
